# Seven New Species of the Genus *Geastrum* (Geastrales, Geastraceae) in China

**DOI:** 10.3390/jof9020251

**Published:** 2023-02-14

**Authors:** Xin Wang, Tolgor Bau

**Affiliations:** Key Laboratory of Edible Fungal Resources and Utilization (North), Ministry of Agriculture and Rural Affairs, Jilin Agricultural University, Changchun 130118, China

**Keywords:** *Geastrum*, morphology, molecular systematics, new species, taxonomy

## Abstract

*Geastrum* belongs to Basidiomycota, Agaricomycetes, Geastrales, and Geastraceae. The genus *Geastrum* exoperidium normally splits at maturity into a characteristic star-like structure. It is a saprophytic fungus with great research significance. Based on morphological observation combined with phylogenetic analysis through ITS and LSU, seven new species of *Geastrum* belong to four sections, viz., Sect. *Myceliostroma*, *Geastrum laneum*; Sect. *Exareolata*, *Geastrum litchi*, *Geastrum mongolicum*; Sect. *Corollina*, *Geastrum pseudosaccatum*, *Geastrum melanorhynchum*, *Geastrum oxysepalum*; and Sect. *Campestria*, *Geastrum microphole*. Illustrated descriptions and the ecological habits of the novel species are provided.

## 1. Introduction

The genus *Geastrum* is a type of gasteroid Basidiomycota, which has been recorded on all continents except Antarctica, mostly in the forest humus layer, although it is occasionally seen on rotten wood or sand and grassland [1]. Some species of *Geastrum* are medicinal fungi with vital research value, and some species are widely used in forestry production practice, as they are able to enhance the absorption function of forest roots and improve the survival rate of afforestation [2]. They can be used as a natural hygrometer based on whether the exoperidium is hygroscopic [2]. As of November 2022, the Index Fungorum recorded more than 100 species.

*Geastrum* was first identified by Persoon (1794) [3]. Then De Toni (1887) proposed dividing the *Geastrum* genus into seven sections based on the morphological characteristics of the peristome, stalk, and exoperidium, viz., Sect. *Columnati*, Sect. *Fornicati*, Sect. *Cupulati*, Sect. *Striati*, Sect. *Fimbriati*, Sect. *Papillati*, and Sect. *Exareolati* [4]. This was later endorsed by Hollós (1903) [5]. Staněk (1958) proposed dividing *Geastrum* into two sections based on differences between the peristomes and whether an encrustation of debris is present, viz., Sect. *Perimyceliata* and Sect. *Basimyceliata* [6]; this was later endorsed by Sunhede (1989) [7]. Section *Basimyceliata* was further revised by Dissing and Lange (1962) [8], based on the integrity of the endoperidium, and subdivided into three subsections and eight species. Ponce (1968) divided the *Geastrum* into one subgenus and six section, viz., Subg. *Geastrum*, Sect. *Geastrum*, Sect. *Basimyceliatum*, Sect. *Myceliostroma*, Sect. *Subepigaea*, Sect. *Trichaster*, and Sect. *Lignicola* [1]. Dörfelt (1985) divided the *Geastrum* genus into four subgenera, viz., Subg. *Trichaster*, Subg. *Geastrum*, Subg. *Pectinata*, and Subg. *Myceliostroma* [9]. In addition, Lloyd (1902) proposed that depending on whether the exoperidium is hygroscopic, this factor can be used as a basis for categorization and *Geastrum* can be divided into two sections, viz., Sect. *Rigidae* and Sect. *Non-rigidae* [10]. Because the *Geastrum* genus has few classification features, its subgenera are mainly distinguished according to morphological features [11]. They are highly divergent due to the different relative value that each author gave to particular morphological features, so it is difficult to group using traditional categorization methods alone, calling for a need to combine the support of molecular data [11,12].

With the development of molecular biology, DNA sequencing has also been used in the taxonomy of the *Geastrum*. Jeppson (2013) first established the phylogeny of the starfish genus and combined morphological and chemical characteristics to record 30 European species [12,13]. Zamora et al. (2013, 2014) constructed a phylogenetic framework based on a combination of morphological characteristics, phenoloxidase activity, and molecular data, dividing the astral genus into 14 sections, viz., Sect. *Campestria*, Sect. *Corollina*, Sect. *Elegantia*, Sect. *Exareolata*, Sect. *Fimbriata*, Sect. *Fornicata*, Sect. *Geastrum*, Sect. *Hariotia*, Sect. *Hieronymia*, Sect. *Myceliostroma*, Sect. *Papillata*, Sect. *Pseudolimbata*, Sect. *Schmidelia*, and Sect. *Trichaster* [14,15,16,17]. New species have been reported on all continents in recent years (Assis et al. 2019; Finy et al. 2021) [18,19].

At present, 27 species of the star genus have been reported in China (Species 2000) and were initially discussed predominantly in illustrative or comprehensive research literature (Teng 1963, Tai 1979, Liu 1984, Yuan et al. 1995, Mao 2000, Li et al. 2003, Zhou 2007, Li et al. 2015) [20,21,22,23,24,25,26,27]. In 2007, Zhou Tong-Xin et al. published a monograph and recorded 16 species. In recent years, our team (Han and Bau 2016) studied the taxonomy of Geastraceae in Jilin Province and found three newly recorded species in China, viz., *G. schweinitzii* (Berk. & M.A. Curtis) Zeller, *G. hungaricum* Hollós, and *G. campestre* Morgan [28]. More recently, Zhou et al. (2021) discovered two new species in the Yanshan Mountains, viz., *G. yanshanense* C.L Hou, Hao Zhou & Jiqi Li and *G. beijingense* C.L Hou, Hao Zhou & Jiqi Li [29].

However, there is still a lot to be studied and explored in the richness of the species resources of the *Geastrum* in China. In our recent investigations on the *Geastrum* from China over the past two years, seven new species were found. They were described in detail and illustrated.

## 2. Material and Methods

### 2.1. Morphological Study

Dried specimens used in this study were deposited at the Herbarium of Mycology of Jilin Agricultural University (HMJAU), China. The methodology and notation used here followed those of Cai et al. (2016) and Cui et al. (2018) [30,31].

Macromorphological descriptions were based on fresh specimens, which were photographed in the field with notes and laboratory supplemental measurements. The color description of the basidiomata was based on Kornerup and Wanscher (1978) [32]. Micromorphological studies were carried out using a light microscope and scanning electron microscope. Dried specimens were used to observe microscopic features. Data of the sections (basidiospores, basidia, capillitial hyphae, and exoperidium) were obtained from dried specimens, which were rehydrated in 5% KOH or stained in Congo red when necessary, and the light microscope (Olympus BX50) was used for the examination of microscopic structures with a high-resolution oil objective lens (1000×). The dimensions of basidiospores are given using a notation in the form ‘a–b’. The dimensions of basidium are given using a notation in the form ‘c–d × e–f’. The dimensions of capillitial hyphae are given using a notation in the form ‘g–h’. The abbreviation [n/m/p] represents n basidiospores measured from m basidiomata of p collections. The range ‘a–b’ means the minimum to the maximum of the diameter. The range ‘c–d’ means the minimum to maximum length, ‘e–f’ and‘g–h’ means the minimum to maximum width. 

For scanning electron microscopy, air-dried samples were mounted on a sample holder covered with double-sided adhesive tape, sprayed with pure gold until fully coated using an ion sputtering instrument IXRF MSP-2S, and observed with a Hitachi SU8010.

### 2.2. DNA Extraction, Amplification and Sequencing

Genomic DNA was extracted from 0.1 to 0.2 mg of dried specimen using a NuClean Plant Genomic DNA kit (CWBIO, Beijing, China) and preserved at −20 °C. The 30 μL PCR reaction system is shown in Table 1. Two molecular markers were investigated, i.e., ITS1F (3′-CTTGGTCATTTAGAGGAAGTAA-5′) and ITS4 (5′-TCCTCCGCTTATTGATATGC-3′), which were used as primers for the internal transcribed spacer (ITS) (White et al. 1990, Gardes et al. 1993) [33,34]. LR0R (5′-ACCCGCTGAACTTAAGC-3′) and LR5 (5′-ATCCTGAGGGAAACTTC-3′) were used for the large subunit of the nuclear ribosomal RNA gene (nrLSU). The PCR procedure for ITS (including 5.8 S) was as follows: initial denaturation at 94 °C for 4 min, followed by 35 cycles at 94 °C for 35 s, 54 °C for 35 s, and 72 °C for 45 s, and a final extension of 72 °C for 10 min. The PCR procedure for nrLSU was as follows: initial denaturation at 94 °C for 4 min, followed by 35 cycles at 94 °C for 1 min, 53 °C for 1 min, 72 °C for 1 min, and a final extension of 72 °C for 10 min. The PCR products were purified and sequenced in Bioengineering (Shanghai) Co., Ltd., China, with the same primers. The newly generated sequences were deposited at GenBank (https://www.ncbi.nlm.nih.gov (accessed on 8 December 2022)). All sequences analyzed in this study were deposited at GenBank and are listed in Table 2.

### 2.3. Phylogenetic Analyses

The new sequences generated in this study were combined with the sequences downloaded from GenBank and outgroups *Schenella pityophila* (Malençon & Riousset) Estrada & Lado and *Sphaerobolus iowensis* (L.B. Walker) Zellerwere used as the outgroups, according to Zamora et al. (2014) and József et al. (2005) [11,35]. Detailed information for these sequences is given in Table 1. After PCR amplification, unidirectional sequencing of ITS products followed. Then, the products of nrLSU were sequenced in a bidirectional sequence and were assembled using a Sequencher 5.4.5 (Gene Codes, Ann Arbor, Michigan, USA). DNA sequences were aligned using MAFFT 7.110 with the G-INI-I option, while the few ambiguously aligned regions of the ITS and nrLSU alignments were removed with Gblocks v.0.91b, by keeping the default settings but allowing all gap positions when not ambiguous and manually adjusted in Sequencher 5.4.5 [36,37].

Maximum likelihood (ML) analysis was performed in RAxML v8.2.4 with GTRGAMMA model [38]. The best tree was obtained by executing 100 rapid bootstrap inferences, and thereafter a thorough search for the most likely tree using one distinct model/data partition with joint branch length optimization. ModelFinder was used to select the best-fit partition model (Edge-linked) using Bayesian information criterion (BIC) [39]. Best-fit model, according to BIC, were the GTR+I+G+F model for the ITS subset (1–620) and the GTR+I+G+F model for the nrLSU subset (621–1584). We used the GTR+I+G+F model. Bayesian Inference phylogenies were inferred using MrBayes 3.2.6; under partition model (two parallel runs, 10,554,300 generations), in which the initial 25% of sampled data were discarded as burn-in, four chains and sampling for every 100th generation four Markov chains (MCMC) were run until the split deviation frequency value was <0.01 [40]. Finally, FigTree version 1.4.3 was used to visualize the phylogenetic trees [41]. Branches that received bootstrap values for maximum likelihood (ML) ≥ 75% and Bayesian inference (BI) ≥ 0.95 were considered as significantly supported.

## 3. Results

### 3.1. Phylogeny

**Figure 1:**
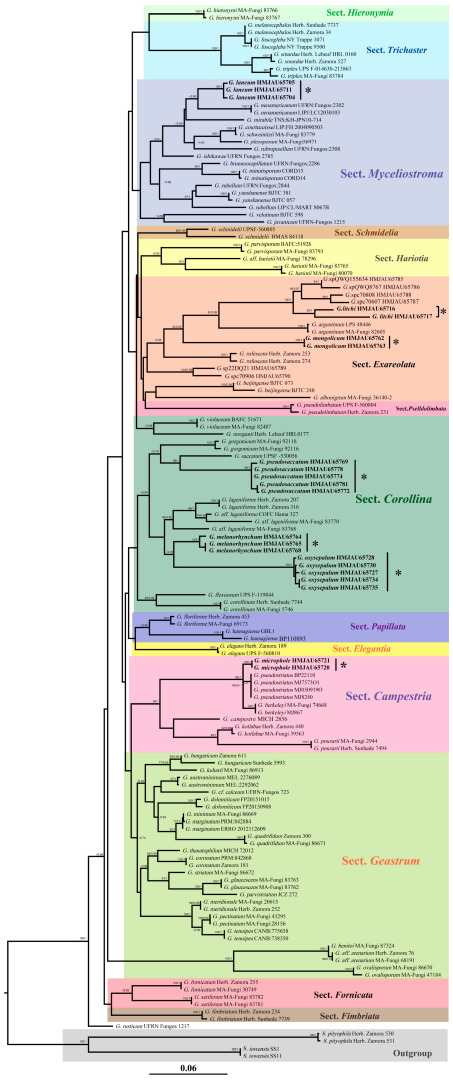
Multi-gene phylogenetic tree obtained from the maximum likelihood analysis (ML). Number above branches are maximum likelihood bootstrap (MLbs) values and Bayesian posterior probability (pp) values. Support values (MLbs > 75%) and posterior probabilities (PP > 0.75) are shown on each branch. The asterisks (*) indicate the position of the new species.

A total of 28 new sequences were generated for this study and with the 144 sequences downloaded from GenBank. In the phylogenetic analysis of the combined dataset (ITS, nrLSU), the aligned lengths of the two gene loci were 606 and 962 base pairs. Bayesian and ML analysis resulted in a same topology. Bayesian analysis has an average standard deviation of split frequencies equal to 0.007893. Only the ML tree is provided in Figure 1; ML bootstrap values (≥75%) and PP (≥0.75) are shown at the nodes.

The constructed phylogenetic tree is similar to the branching structure given in Zamora et al. (2014). The difference is that the position of the sections in the phylogenetic tree is slightly different. The species in the sections are the same. This phylogenetic tree is different from the branch support rate of the phylogenetic tree established by Zamora. However, these differences are allowed to exist and do not affect the position of the species on the phylogenetic tree. 

The phylogenetic tree shows seven new species in four sections. In Sect. *Myceliostroma*, a new species *G. laneum*, with high support values for all specimens, was found (PP = 1.00, MLbs = 99%) and formed a sister branch with a higher support value (PP = 0.95) with *G. neoamericanum* J.O. Sousa, Accioly, M.P. Martín & Baseia. At the same time, this new species and other species in the section can also be well distinguished on the phylogenetic tree.

There are two new species in Sect. *Exareolata*. One is *G. litchi* with high support values for all specimens in this section (PP = 1.00, MLbs = 95%) and formed a sister branch with *G. argentinum Speg.* with a higher support value (PP = 0.99, MLbs = 82%). There is a Long Branch Attraction in the genus of *Geastrum*. There are also intraspecific variation in *G. litchi*. Therefore, the two specimens representing *G. litchi* have a relatively long phylogenetic distance compared with other species in this section. The other is *G. mongolicum* with high support values for all specimens in this section (PP = 1.00, MLbs = 100%) and with *G. rufescens* Pers. formed a sister branch with a higher support value (PP = 0.98, MLbs = 64%). These two new species and other species in the section can also be well distinguished on the phylogenetic tree.

There are three new species in Sect. *Corollina*. The first is *G. pseudosaccatum* with high support values for all specimens in this section (PP = 1.00, MLbs = 99%) and formed a sister branch with a higher support value (PP = 1.00, MLbs = 89%) with *G. saccatum* Fr. The second is *G. melanorhynchum* with high support values for all specimens in this section (PP = 1.00, MLbs = 100%), and the third is *G*. *oxysepalum* with high support values for all specimens in this section (PP = 1.00, MLbs= 100%). The last two new species are sister taxa to each other and form a clade with higher support values (PP = 0.78). The last two new species with *G. lageniforme* Vittad formed a sister branch with a higher support value (PP = 0.99, MLbs = 84%). These three new species and other species in this section can also be well distinguished on the phylogenetic tree.

In Sect. *Campestria*, a new species *G. microphole* with high support values for all specimens in this section (PP = 1.00, MLbs = 100%) and with *G. pseudostriatum* Hollós formed a sister branch with a higher support value (PP = 1.00, MLbs = 100%). At the same time, this new species and other species in this section can also be well distinguished on the phylogenetic tree. 

Zamora et al. (2014) divided the genus into 14 branches based on morphological, chemical, and molecular phylogenetic data, and explained in detail the relationship between the branches they found; all of them were strongly to moderately supported when the results from the three different phylogenetic analyses were combined [11]. This paper mainly uses molecular data to construct a phylogenetic tree, showing the division of 14 parts as shown in (Figure 1). As noted above, although tree topologies were almost identical between the ML and Bayesian trees, they are different in support rate, which may be related to their respective calculation methods. The low branch support rate may be due to the long branch attraction effect. Because of the lack of other species that have not been found on the branch, the branch length and support rate are affected; this is also the reason why some sections are not very stable, and some sections will have multiple sources. These problems need to be studied through a large number of field collections in the future. However, these differences do not affect the overall branching stability, nor do they affect the branching stability of new species (Figure 1).

### 3.2. Taxonomy

**Figure 2:**
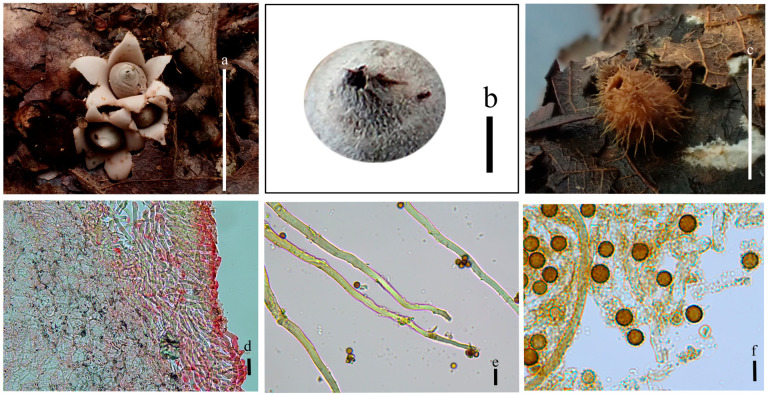
*Geastrum laneum* (HMJAU65704, HMJAU65713, HMJAU65706). Bars: (**a**) basidiomata = 1 cm; (**b**) peristome = 1 mm; (**c**) unexpanded basidiomata = 1 cm; (**d**) exoperidium three layers = 10 μm; (**e**) capillitial hyphae = 10 μm; (**f**) basidiospores = 5 μm.

**Figure 3:**
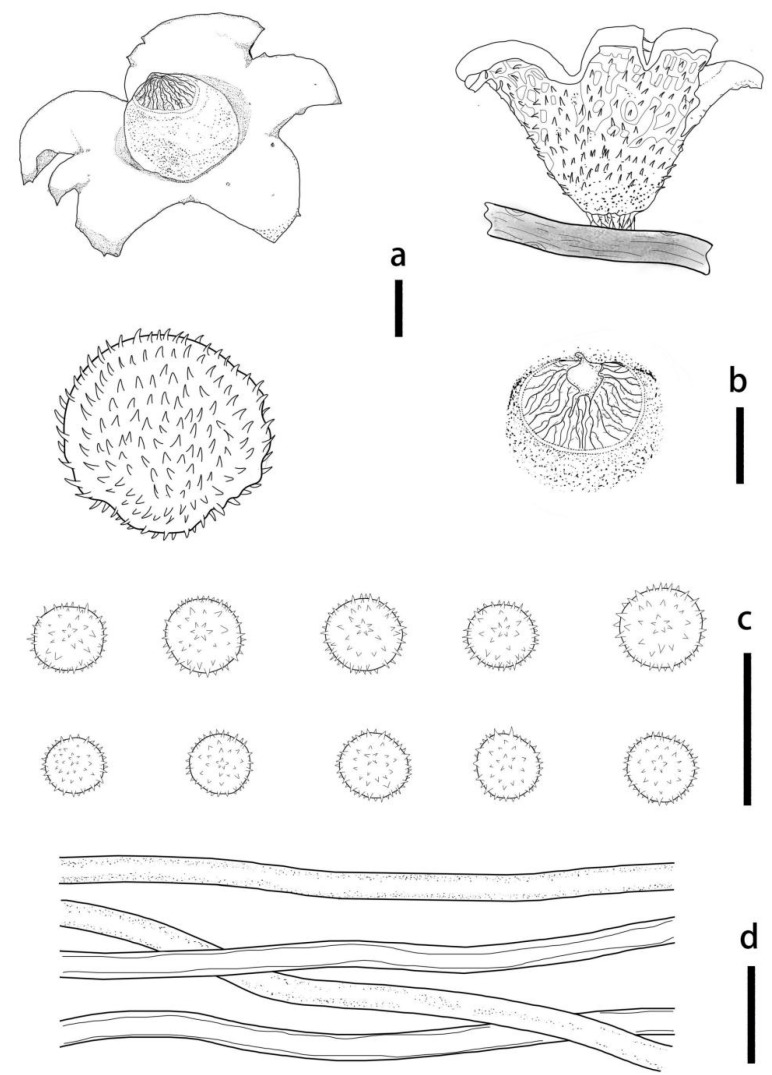
*Geastrum laneum,* Bars: (**a**) basidiomata = 1 cm; (**b**) peristome = 1 mm; (**c**) basidiospores = 5 μm; (**d**) capillitial hyphae = 10 μm.

***Geastrum laneum*** T. Bau & X. Wang, **sp. nov.** (Figure 2 and Figure 3).

MycoBank no: MB846867.

Diagnosis: Differs from *G. mirabile* (Mont.) E., Fisch, in terms of the mycelial tufts, the latter expanded basidiomata has a mycoderm at the base, the mycelial layer is not encrusted with debris; peristome fibrillose; basidiospores displays a delicately warry or columnar process [26].

Type: China, Anhui Province: Zipeng Mountain Forest Park, Hefei City, 31°43′ N, 117°00′ E, lat. 77.42 m, 13 July 2021, Qingqing Dong, 21713DQQ11 (holotype, HMJAU65704).

Etymology: ‘*laneum*’ refers to its mycelial layer visible coarse short villus in a felted form.

Description: Unexpanded basidiomata, 3–10 mm in size, with a few white (5A1) mycoderma. Expanded basidiomata small, 4.5–9.5 mm. The exoperidial disc has a diameter of 1.5–7 mm. Exoperidium: shallowly saccate, deeply saccate, dehiscence often less than halfway down, at maturity splits into 5–7 lobes, lobes 1–7 mm wide, extremely narrow at the apex and blunt, rolled outwards to underneath exoperidial disc, with occasional spreading, soft and thin when dry. Pseudoparenchymatous layer: smooth surface, camel (6D4), mostly contracted along the margin of lobes or broken at the base of cleft, not deciduous, aseptic collar, thinner when dry. Fibrous layer: yellowish white (4A2) to yellowish grey (4B2), tightly attached to the mycelial layer. Mycelial layer: Henna (7E8), visible coarse short villus in a felted form, encrusted with debris.

Endoperidial body: globular or ovate, 2–7 mm in diameter, projecting apically or extending into a beak, 0.5–1.0 mm length, sessile, without an apophysis. Endoperidium: brownish grey (6E2), with a smooth surface and greyish villus visible under the dissecting microscope. Peristome: broad-conical, silkily fibrillose, darker in color than the endoperidium, distinctly delimited.

Basidiospores: spherical, 2.5–3.9 μm in diameter, yellowish brown to dark brown in contact with 5% KOH solution, surface with delicately echinulate, length 0.3–0.7 μm, non-starchy, echinulate under scanning electron microscope. Capillitial hyphae: up to 0.4–5.8 μm in diameter, thick-walled, tawny, without branches, surface with yellow crusts, and sparse surface debris. Exoperidium: 357.4–532.5 µm thick, the pseudoparenchymatous layer formed of pseudoparenchymatous of angular cell structured, 19.3–52.6 × 13.3–24.1 μm; fibrous layer formed of thick-walled interlacing filament tissue, 2.5.1–5.0 μm; the mycelium layer formed of thick-walled 2.7–4.2 μm diam hyphae.

Additional specimens examined: China, Anhui Province: Siddingshan, Hefei, alt. 174 m, 6 July 2020, Liyang Zhu, Z706WX12 (HMJAU65713), same location, 6 July 2020, Liyang Zhu, Z207610 (HMJAU65712); China, Jiangsu Province: Zijinshanzhuang, Nanjing, alt.445 m, 19 July 2020, Liyang Zhu, Z2071914w (HMJAU65711), same location, 10 July 2021, Zhu Liyan, z21071006(HMJAU65709); China, Jiangsu Province: Linggu Temple Scenic Area, Zijinshan, Nanjing, alt.425 m, 26 July 2020, Liyang Zhu, Z20726W11 (HMJAU65714); China, Zhejiang Province: Taizhou6 June 2021, alt.14 m, Jingli Wang, wjl21060619 (HMJAU65715); China, Anhui Province: Zipeng Mountain Forest Park, Hefei City, alt. 77.42 m, 8 July 2020, Liyang Zhu, Z200708 (HMJAU65708), same location, 13 July 2021, Liyang Zhu, Z21071319 (HMJAU65706), 13 July 2021, Liyang Zhu, Z21071320 (HMJAU65707), 13 July 2021,Zhu Liyang, z21071312 (HMJAU65705); China, Jiangsu Province: purple mountain, Nanjing, alt. 448.9 m, July 16, 2020, Liyang Zhu, Z20716WX7 (HMJAU65710).

Habitat: Grows on crustacean decay or on dead branches.

Distribution: Anhui Province, Jiangsu Province, Zhejiang Province, China.

**Figure 4:**
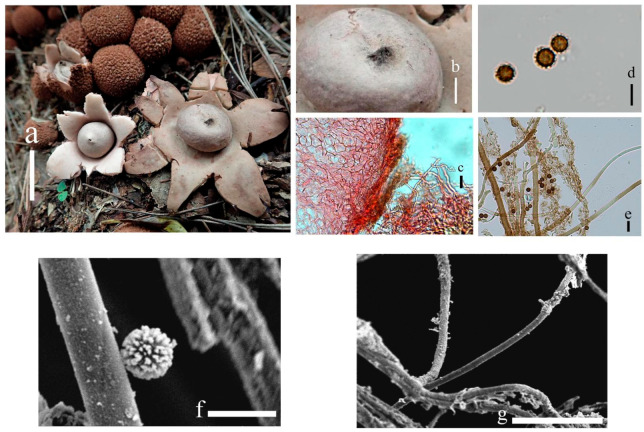
Geastrum litchi (HMJAU65716, HMJAU65717), Bars: (**a**) basidiomata = 2 cm; (**b**) peristome = 2 mm; (**c**) exoperidium = 20 μm; (**d**) basidiospores = 5 μm; (**e**) capillitial hyphae = 10 μm; (**f**) = 5 μm; (**g**)= 10 μm.

**Figure 5:**
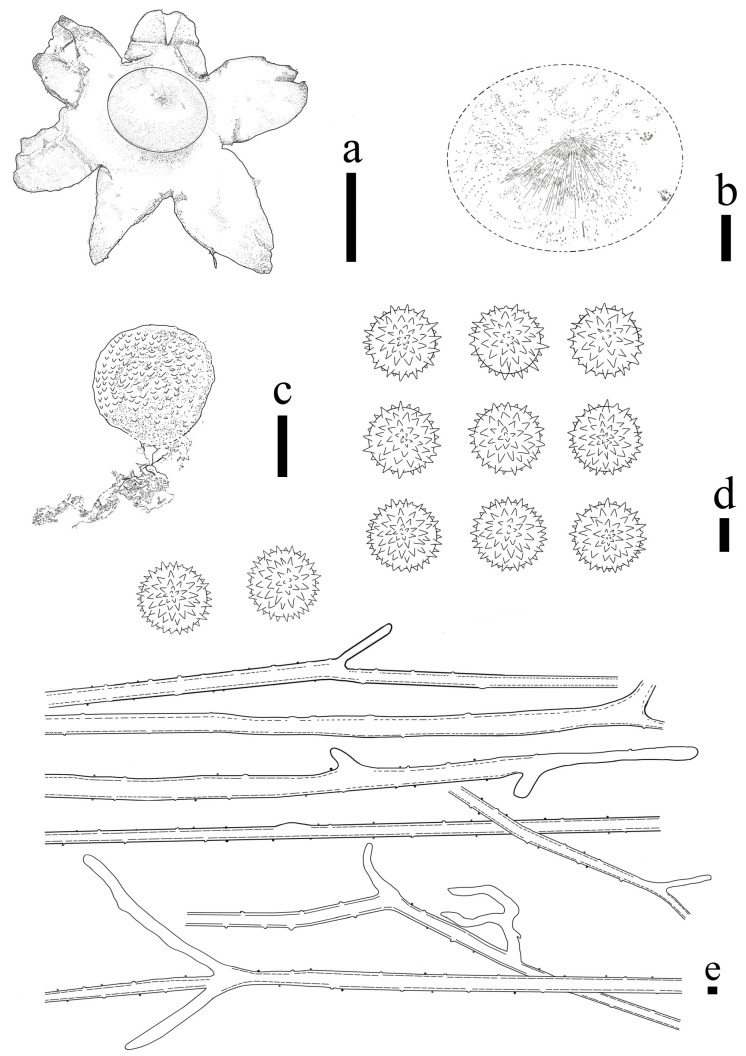
*Geastrum litchi*, Bars: (**a**) basidiomata = 2 cm; (**b**) peristome = 2 mm; (**c**) unexpanded basidiomata = 1 cm; (**d**) basidiospores = 1 μm; (**e**) capillitial hyphae = 1 μm.

***Geastrum litchi*** T. Bau & X. Wang, **sp.nov.** (Figure 4 and Figure 5).

MycoBank no: MB846868.

Diagnosis: Differs from *G. litchiforme* Desjardin & Hemmes by the pseudoparenchymatous layer, the latter frequently forming a collar around the base of the endoperidium; peristome undelimited, dark brown overall, easily separable from the expanding basidiome; odor of crushed unexpanded basidiomata strong, similar to that of bok choy [42].

Type: China, Guangdong Province: Danxia Mountain, Yunguan City, 113°36′25″ E to 113°47′53″ E, 24°51′48″ N to 25°04′12″ N, alt. 117 m, 4 June 2019, T. Bau, T19060401 (holotype, HMJAU65716).

Etymology—‘*litchi*’ refers to the mycelial layer surface covered with reddish-brown small pyramidal tufts of villus that produce an areolate pattern similar to the surface of a lychee fruit.

Description: Unexpanded basidiomata dark reddish brown (8E6), 0.9–2.3 cm in diameter, white mycelial tufts, and scent of light chocolate. Expanded basidiomata small to medium sized, 1.6–2.4 cm in diameter. Exoperidium: shallowly to deeply saccate, splits into 5–7 lobes at maturity, lobes 0.5–1.1 cm wide, tapered at the front end, rays nonhygroscopic. Pseudoparenchymatous layer: smooth surface, brownish grey (8C2), contracted along margin of lobes or at base of lobes breaking, easily exfoliation, aseptic collar. Fibrous layer: grey (8B1), tightly attached to the mycelial layer. Mycelial layer: surface covered with small reddish brown (8E6) pyramidal tufts of villus that produce an areolate pattern similar to the surface of a lychee fruit, not easily dislodged, not encrusted with debris.

Endoperidial body: globular, 1.2–1.4 cm in diameter, projecting apically or extending into a beak, 0.1–0.2 cm length, sessile, without an apophysis. Endoperidium: brownish grey (8D2) with pale powder, with a smooth surface and greyish villus visible under the dissecting microscope. Peristome: broad-conical, silky fibrillose, shallower or darker in color than the endoperidium, undelimited.

Basidiospores: spherical, 2.8–4.1 μm in diameter, yellowish brown to dark brown in contact with 5% KOH solution, surface with short columnar process, 0.4–0.9 μm length, non-starchy, columnar process under scanning electron microscope. Capillitial hyphae up to 2.0–7.0 μm in diameter, thick-walled, pale brown to yellowish brown, few unbranched, many with short branches, occasionally long branches, with denser surface debris. Exoperidium: 898–1136 µm thick, the pseudoparenchymatous layer formed of the pseudoparenchymatous in an angular cell structure, 15.5–39.4 × 8.9–21.2 μm; fibrous layer formed of thick-walled interlacing filament tissue, 2.7–4.9 μm; the mycelium layer formed of thin-walled hyphae diam 4.1–13.2 μm.

Additional specimens examined: China, Guangdong Province: Danxia Mountain, Yunguan City, alt. 117 m,4 June 2019, Tolgor bau, T19060402(HMJAU65717).

Habitat: Broad-leaved forest ground.

Distribution: Guangdong Province, China.

**Figure 6:**
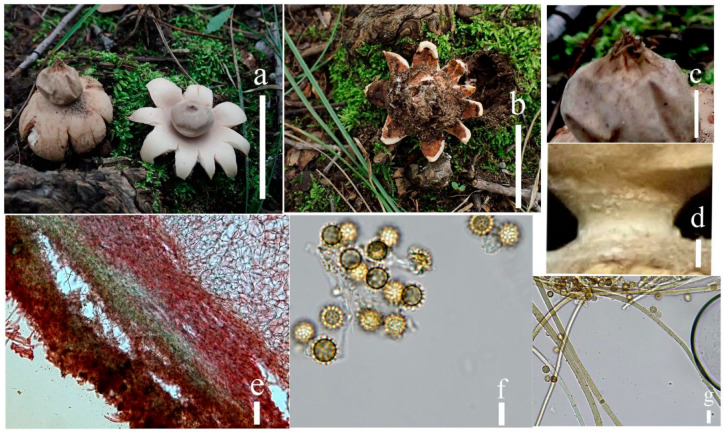

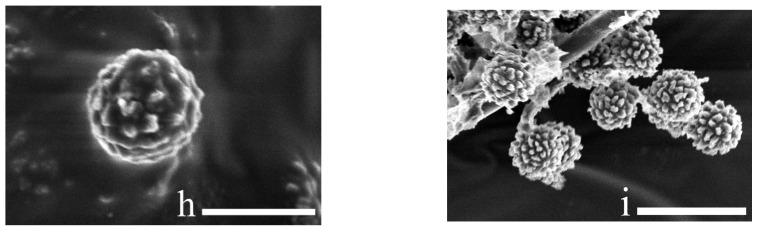
*Geastrum mongolicum* (HMJAU65762, HMJAU65763). Bars: (**a**,**b**) basidiomata = 2 cm; (**c**) peristome = 1 mm; (**d**) stalk = 1 mm; (**e**) exoperidium = 50 μm; (**f**) basidiospores = 5 μm; (**g**) capillitial hyphae = 10 μm; (**h**) = 5 μm; (**i**) = 10 μm.

**Figure 7:**
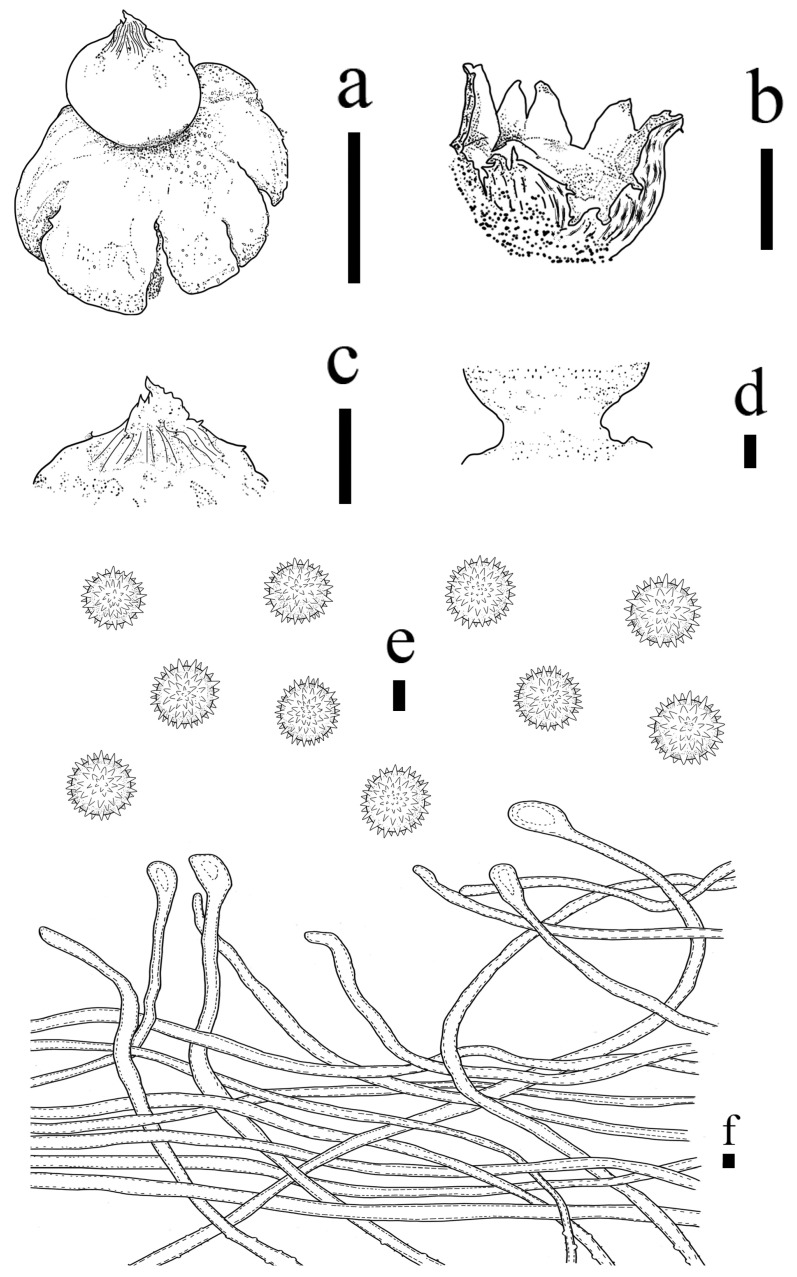
*Geastrum mongolicum*, Bars: (**a**,**b**) basidiomata = 2 cm; (**c**) peristome = 1 mm; (**d**) stalk = 1 mm; (**e**) basidiospores = 2 μm; (**f**) capillitial hyphae = 2 μm.

***Geastrum mongolicum*** T. Bau & X. Wang, **sp.nov.** (Figure 6 and Figure 7).

MycoBank no: MB846869.

Diagnosis: Differs from *G. rufescens* Pers. by the pseudoparenchymatous layer, the latter initially pale pink, with age pinkish to reddish brown, frequently forming a collar around the base of the endoperidium; peristome undelimited, color same as endoperidium, usually without an apophysis, basidiospores with columnar process or rough warry or delicate warry, capillitial hyphae with a dense cover of surface debris [12,13].

Type: China, Inner Mongolia Autonomous Region: Wudantala Forest Farm, Horqin Left Wing Rear Banner, Tongliao, N 43°02′30″–42°57′20″, E 122°40′50″–122°49′00″, alt. 336 m, 5 August 2021, T. Bau & X. Wang, WX20218525 (holotype, HMJAU65762).

Etymology: ‘*mongolicum*’ refers to its occurrence in Inner Mongolia Autonomous Region, China.

Description: Expanded basidiomata 1.9–2.2 cm, exoperidial disc 2–2.5 cm in diameter. Exoperidium: arched, deep saccate, dehiscence often greater than halfway down, at maturity splits into 7–10 lobes, lobes 0.2–0.6 cm wide. Pseudoparenchymatous layer: thick when fresh, greyish brown (8D3) or brownish grey (8C2,8D2), surface with transparent granular crystals sparse, mostly longitudinally fissured when dry, easily detached, aseptic collar. Fibrous layer: white (8A1), tightly attached to the mycelial layer. Mycelial layer: reddish brown (8E7), rough, wrinkled, easily dislodged, encrusted with debris.

Endoperidial body: globular, 0.8–1.4 cm in diameter, projecting apically or extending into a beak, 0.1–0.2 cm length, stipitate 0.1–0.2 cm length, apophysis. Endoperidium: greyish brown (8E3) or brownish grey (8E2), with a smooth surface and greyish villus visible under the dissecting microscope. Peristome: broad-conical, silky fibrillose, color lighter than or equal to the endoperidium, undelimited, occasional raised.

Specialized mycelium: end enlarged with oil droplets, utriform, thick-walled. Basidiospores: spherical, 4.1–4.5 μm in diameter, brown in contact with 5% KOH solution, surface with delicately echinulate, 0.3–1.0 μm length, non-starchy, echinulate under scanning electron microscope. Capillitial hyphae: up to 1.7–4.2 μm in diameter, thick-walled, brownish yellow, unbranched, smoother wall, with sparse surface debris. Exoperidium: 485–682 µm thick, the pseudoparenchymatous layer formed of the pseudoparenchymatous of angular cell structured, 27.3–45.2 × 15.9–32.4 μm; fibrous layer formed of thin-walled interlacing filament tissue, 2.4–4.7 μm; the mycelium layer formed of thick-walled hyphae diameter 2.3–4.4 μm.

Additional specimens examined—China, Inner Mongolia Autonomous Region: Wudantala Forest Farm, Horqin Left Wing Rear Banner, Tongliao, alt. 336 m, 5 August 2021, T. Bau & X. Wang, WX20218526 (HMJAU65763).

Habitat: Saprophytism on moss layer scattered under *Quercus mongolica* and *Acer pictum* in sandy terrain.

Distribution: Inner Mongolia Autonomous Region, China.

**Figure 8:**
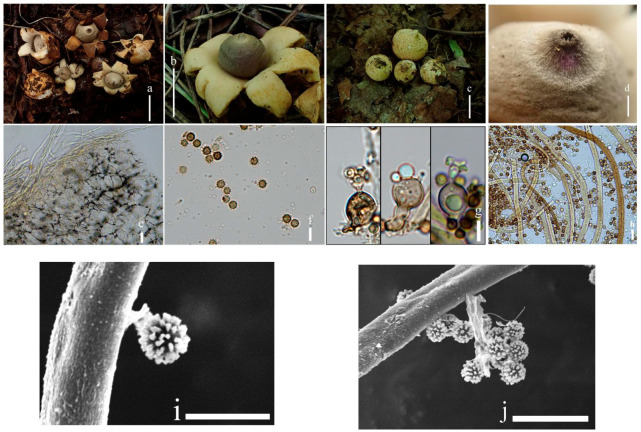
*Geastrum pseudosaccatum* (HMJAU65769, HMJAU65780, HMJAU65784), Bars: (**a**) basidiomata = 2 cm; (**b**) basidiomata = 1 cm; (**c**) unexpanded basidiomata = 1 cm; (**d**) peristome = 1 mm; (**e**) exoperidium = 20 μm; (**f**) basidiospores = 5 μm; (**g**) basidia = 5 μm; (**h**) capillitial hyphae = 10 μm; (**i**) = 5 μm; (**j**) = 10 μm.

**Figure 9:**
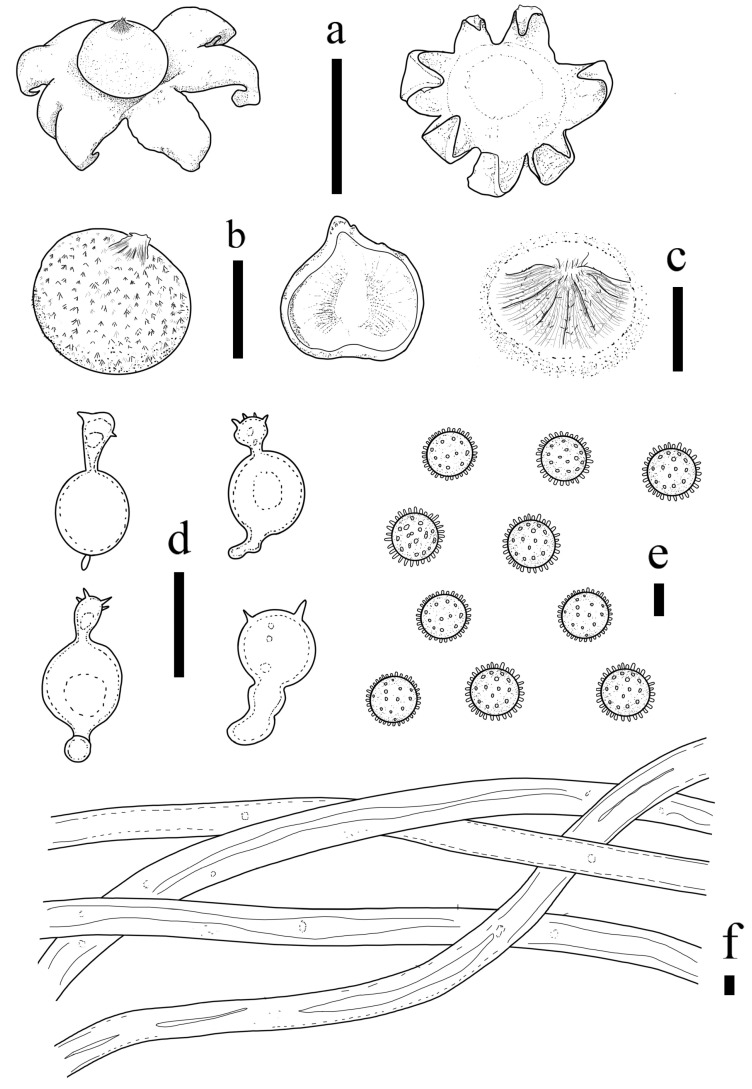
*Geastrum pseudosaccatum*, Bars: (**a**) basidiomata = 2 cm; (**b**) peristome = 1 mm; (**c**) peristome = 1 mm; (**d**) basidia = 10 μm; (**e**) basidiospores = 2 μm; (**f**) capillitial hyphae = 1 μm.

***Geastrum pseudosaccatum*** T. Bau & X. Wang **sp.nov.** (Figure 8 and Figure 9).

MycoBank no: MB846870.

Diagnosis: Differs from *G. saccatum* Fr. by the lobes. Unexpanded basidiomata smooth surface, at maturity splits into (3-)5~8(-10) lobes, pseudoparenchymatous layer 2 mm thick when fresh, mycelial layer with fine villi under dissecting microscope, unseen basidia under the microscope [26].

Type: China, Jilin Province: Liuxian Line, Liuhe County, Tonghua City, 41°54′–42°35′ N, 125°17′–126°35′ E, alt. 482 m, 10 August 2022, X. Wang, 22wx2090810 (holotype, HMJAU65769).

Etymology: ‘*pseudo*’ means false and ‘*saccatum*’ means it has an exoperidium form. Morphologically, this species is similar to *G. saccatum* Fr.

Description: Unexpanded basidiomata onion-shaped, 1.2–1.9 cm width, olive brown (4E5), clustered villi on the surface. Expanded basidiomata are mostly small to medium sized, 1.2–3.7 cm. Exoperidium: shallowly saccate, deep saccate, arched, dehiscence less than or greater than halfway down, at maturity splits into 5–9 lobes, lobes 0.2–1.6 cm wide, lobes mostly rolled outward to under the outer exoperidial disc. Pseudoparenchymatous layer: smooth surface, fresh when thin, reddish brown (8DE) or greyish yellow (4B3), contracted along margin of lobes, aseptic collar. Fibrous layer: reddish white (8A2), tightly attached to the mycelial layer. Mycelial layer: yellowish brown (5E7), smooth surface, sparse fine villi visible under dissecting microscope, easily dislodged, not encrusted with debris.

Endoperidial body: globular, 0.6–2.1 cm in diameter, projecting apically or extending into a beak, 0.1–0.5 cm length, sessile, without an apophysis. Endoperidium: brownish grey (8F2) or yellowish brown (5E4), with a smooth surface and greyish villus visible under the dissecting microscope. Peristome: broad-conical, same as or darker in color than the endoperidium, silkily fibrillose. Peristomal: ringed with a distinctly bulge.

Basidiospores: spherical, 2.6–3.0 μm in diameter, yellowish brown to dark brown in contact with 5% KOH solution, surface with short columnar process, 0.4–0.6 μm length, non-starchy, columnar process under the scanning electron microscope. Basidia: 14.4–19.7 × 9.1–11.4 µm, pale tan, clavate, pyriform to sublageniform, 2(4)-sporde, thick-walled, smooth, sometimes with septum when mature, base flexural stipitate, 1.8–7.9µm length. Capillitial hyphae: up to 3.5–7.1 μm in diameter, thick-walled, pale brownish or tan, unbranched, smooth wall, with sparse surface debris. Exoperidium: 480–571µm thick, the pseudoparenchymatous layer formed of pseudoparenchymatous of angular cell structured, 10.7–43.3 × 6.3–21.9 μm; fibrous layer formed of thin-walled interlacing filament tissue, 2.8–4.3 μm length; the mycelium layer formed of thin-walled hyphae diam 2.9–4.9 μm length.

Additional specimens examined: China, Jilin Province: Qingling, Jiaohe City, alt. 460 m, 24 July 2022, Shien Wang, E220705 (HMJAU65770); China, Jilin Province: Dongshan Park, Panshi City, alt. 411 m, 8 August 2022, Lisong Mu, m137 (HMJAU65771); China, Jilin Province: Lianhua Mountain Primitive Forest Park, Panshi City, alt. 453 m, 9 August 2022, Fang Guo, gf809182 (HMJAU65772), same location, 9 August 2022, X. Wang, 22wx2070809 (HMJAU65773), August 12,2022, X. Wang, 22wx2290812 (HMJAU65774); China, Jilin Province: Liuxian Line, Liuhe County, Tonghua City, alt. 482 m, 10 August 2022, Fang Guo, gf22810183 (HMJAU65775), same location, 10 August 2022, Lisong Mu, m244 (HMJAU65776), 10 August 2022, X. Wang, 2281003w (HMJAU65777); China, Jilin Province: Luotong Mountain, Liuhe County, Tonghua City, alt. 488 m, X. Wang, 11 August 2022, 2281109w (HMJAU65778), same location, 11 August 2022, X. Wang, 22wx2120811 (HMJAU65779); 11 August 2022, Fang Guo, gf22811187 (HMJAU65784); China, Jilin Province: Sanxianjia National Forest Park, Liuhe County, Tonghua City, alt. 666 m, 12 August 2022, X. Wang, 22wx2330812 (HMJAU65780), same location, 12 August 2022, X. Wang, 2281211w (HMJAU65782); China, Jilin Province: Longtanshan Heritage Park, Jilin City, alt. 251 m, 12 August 2022, Siying Li, L58 (HMJAU65781); China, Jilin Province: Yuhuangshan, Tonghua City, alt. 521 m, 14 August 2022, Lisong Mu, m264 (HMJAU65783).

Habitat: Scattered on the humus layer of Pinus thunbergii, Larix gmelinii, Quercus mongolica, Juglans mandshurica.

Distribution: Jilin Province, China.

**Figure 10:**
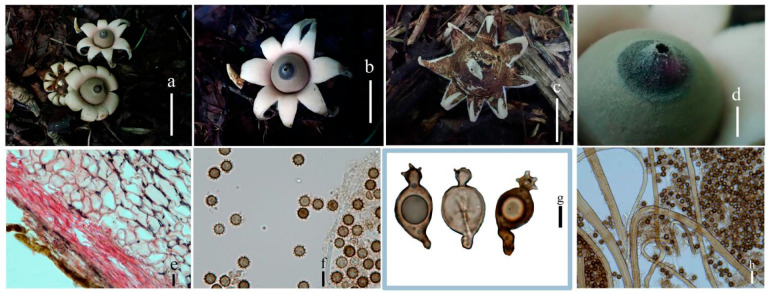

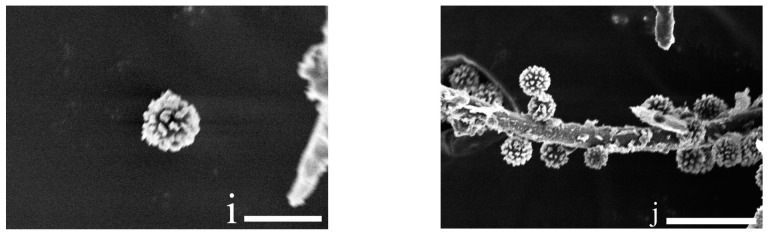
*Geastrum melanorhynchum* (HMJAU65764, HMJAU65767), Bars: (**a**–**c**) basidiomata = 2 cm; (**d**) peristome = 1 mm; (**e**) exoperidium = 50 μm; (**f**) basidiospores = 5 μm; (**g**) basidia = 5 μm; (**h**) capillitial hyphae = 10 μm; (**i**) = 5 μm; (**j**) = 10 μm.

**Figure 11:**
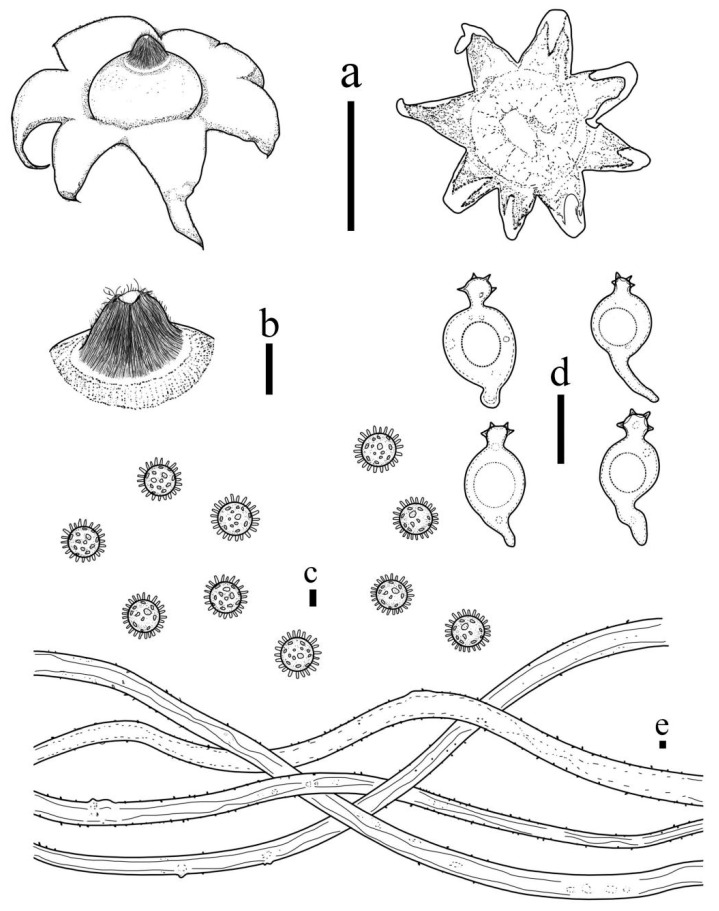
*Geastrum melanorhynchum*, Bars: (**a**) basidiomata = 2 cm; (**b**) peristome = 1 mm; (**c**) basidiospores = 2 μm; (**d**) basidia = 10 μm; (**e**) capillitial hyphae = 1 μm.

***Geastrum melanorhynchum*** T. Bau & X. Wang, **sp.nov.** (Figure 10 and Figure 11).

MycoBank no: MB846871.

Diagnosis: Differs from *G. morganii* Lloyd by the pseudoparenchymatous layer, the latter sandy earthy color with dark olive etc; frequently forms a collar around the base of the endoperidium; peristome undelimited, color same as endoperidium, irregularly sulcate; basidiospores columnar process, a few rough warry [12,13,26]. Differs from *G. lageniforme* Vittad. by the pseudoparenchymatous layer, the latter dark brown, peristome silky fibrillose, basidiospore delicately warry [13].

Type: China, Jilin Province: Jiaohe, Qianjin Experimental Forestry Farm, N 43°51′–44°05′, E 127°31′–127°51′, alt. 460 m, 23 July 2022, X. Wang, 22wx1110723 (holotype, HMJAU65764).

Etymology: ‘*melanorhynchum*’ refers to its peristome being of a darker color than endoperidium, i.e., black, and ‘melano’ means ‘melanidus’ and ‘rhynchus’ means ‘rhynchophorus’.

Description: Expanded basidiomata are mostly small to medium sized, 1.2–3.2 cm. Exoperidium: shallowly saccate, arched, dehiscence often greater than halfway down, at maturity splits into 7–9 lobes, lobes 0.4–1.3 cm wide, lobes long and mostly rolled outward to under the outer exoperidial disc, extremely narrow at the apex. Pseudoparenchymatous layer: smooth surface, reddish grey (9B2) or brownish grey (9B3), contracted along margin of lobes or falling off at base of lobes without breaking, aseptic collar. Fibrous layer: white (9A1), tightly attached to the mycelial layer. Mycelial layer: reddish brown (8E7), felt surface, not easily dislodged, not encrusted with debris.

Endoperidial body: globular, 0.6–2.0 cm in diameter, projecting apically or extending into a beak, 0.2–0.5 cm length, sessile, without an apophysis. Endoperidium: greyish brown (9E3), with a smooth surface and greyish villus visible under the dissecting microscope, gleba black. Peristome: broad-conical, fibrillose, darker in color than the endoperidium, with a distinctly ringed peristomal, occasional raised.

Basidiospores: spherical, 3.5–3.9 μm in diameter, yellowish brown to dark brown in contact with 5% KOH solution, surface with long columnar process, length 0.8–1.1 μm, non-starchy, columnar process under scanning electron microscope. Basidia: 14.4–19.7 × 9.1–11.4 µm, brown, clavate, sublageniform, 4-sporde, thick-walled, smooth, with large oil droplets and vacuoles, base flexural stipitate, stalk 1.6–5.9 µm length. Capillitial hyphae: up to 3.2–8.0 μm in diameter, thick-walled, brown to tan, mostly unbranched, occasionally branched, with sparse surface debris. Exoperidium: 251.6–558.4 µm thick, the pseudoparenchymatous layer formed of pseudoparenchymatous of angular cell structured, 18.9–37.1 × 13.3–24.1 μm; fibrous layer formed of thin-walled interlacing filament tissue, 3.1–5.6 μm; the mycelium layer formed of thin-walled hyphae diameter 2.8–5.3 μm.

Additional specimens examined: China, Jilin Province: Jiaohe, Qianjin Experimental Forestry Farm, alt. 460 m, 23 July 2022, Fang Guo, gf22723101 (HMJAU65765), same location, 23 July 2022, Lisong Mu, m120 (HMJAU65766); 23 July 2022, Liyang Zhu, z22072338 (HMJAU65767); China, Jilin Province: Sanxianjia National Forest Park, Liuhe County, alt. 666 m, 12 August 2022, X. Wang, 22wx2280812 (HMJAU65768)

Habitat: Scattered on the humus layer of *Fraxinus mandshurica* and *Juglans mandshurica*.

Distribution: Jilin Province, China.

**Figure 12:**
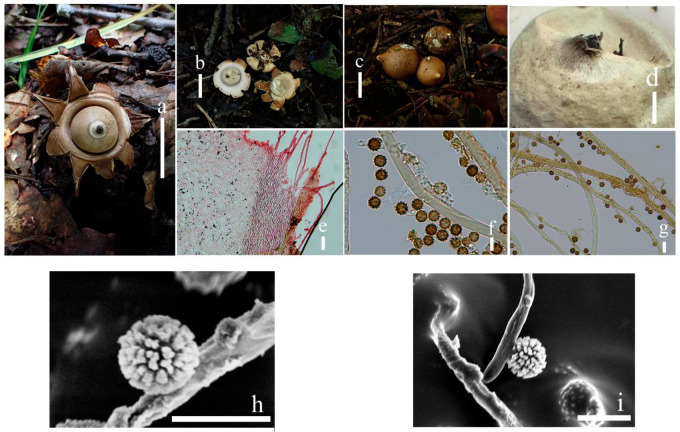
*Geastrum oxysepalum* (HMJAU65727, HMJAU65731, HMJAU65736), Bars: (**a**,**b**) basidiomata = 1 cm; (**c**) unexpanded basidiomata = 1 cm; (**d**) peristome = 1 mm; (**e**) exoperidium = 50 μm; (**f**) basidiospores = 5 μm; (**g**) capillitial hyphae = 10 μm; (**h**) = 5 μm; (**i**) = 5 μm.

**Figure 13:**
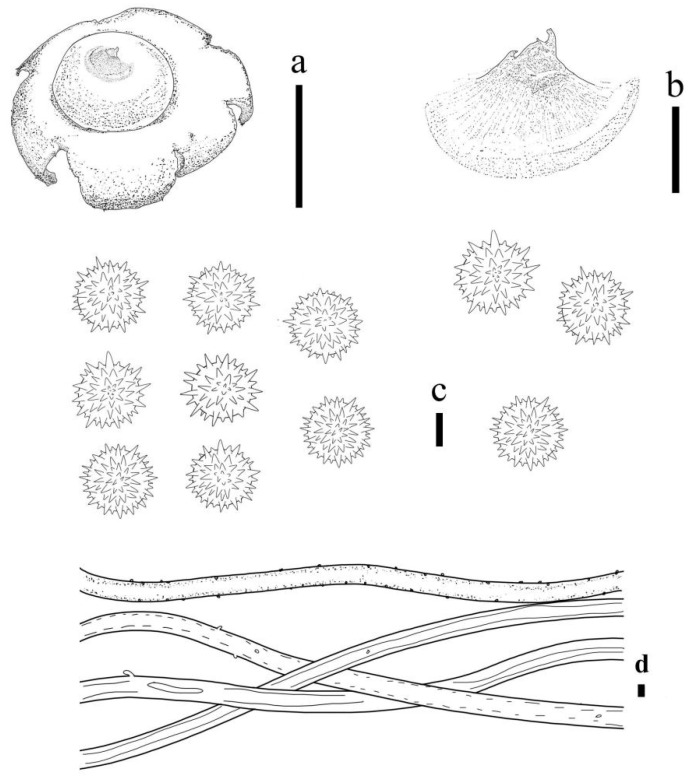
*Geastrum oxysepalum, * Bars: (**a**) basidiomata = 1 cm; (**b**) peristome = 1 mm; (**c**) basidiospores =2 μm; (**d**) capillitial hyphae = 1 μm.

***Geastrum oxysepalum*** T. Bau & X. Wang, **sp.nov.** (Figure 12 and Figure 13).

MycoBank no: MB846872.

Diagnosis: Differs from *G.lageniforme* Vittad. by the exoperidium, the latter lobe surface with radial stripes, terminal tip of lobe, dark brown pseudoparenchymatous layer, endoperidium with a distinctly delimited and no bulge [13]. Silky fibrillose peristome, basidio spore delicately warry. Differs from *G. saccatum* Fr. by the pseudoparenchymatous layer, the latter breaks but does not form collar, mycelial layer with fine villi under dissecting microscope, endoperidium with a distinctly delimited and no bulge [26].

Type: China, Jilin province: Jingyuetan National Forest Park, Changchun City, 43°52′ N, 125°21′ E, alt. 306 m, 18 September 2021, X. Wang, wx2191812 (holotype, HMJAU65727).

Etymology: ‘*oxysepalum* ’ means pointed sepals, also known as acute sepals, and refers to its exoperidium after drying extremely narrow at the apex.

Description: Unexpanded basidiomata brown (6E6,6E5), 1.3–1.6 cm, white mycelial tufts. Expanded basidiomata are 1.2–1.7 cm. The exoperidial disc has a diameter of 0.6–1.5 cm. Exoperidium: shallowly saccate, arched, dehiscence often greater than halfway down, at maturity splits into 5–8 lobes, lobes 0.2–0.7 cm wide, after drying to an extremely narrow point at the apex. Pseudoparenchymatous layer: smooth surface, fresh when thick, yellowish white (4A2), brown (6E5), brownish grey (8D2) or greyish brown (8D3), contracted along margin of lobes, dried thin, brown to dark brown, not falling off, aseptic collar. Fibrous layer: white (6A1), greenish grey (1B2), tightly attached to the mycelial layer. Mycelial layer: olive brown (4D4), not easily dislodged, not encrusted with debris.

Endoperidial body: globular, 0.6–1.3 cm in diameter, projecting apically or extending into a beak, 0.1–0.3 cm length, sessile, without an apophysis. Endoperidium: brown (6E4), greyish green (1C3), with a smooth surface and greyish villus visible under the dissecting microscope. Peristome: broad-conical, fibrillose, darker in color than the endoperidium, with a slightly raised, non-constant peristomal ring.

Basidiospores: spherical, 2.7–3.9 μm in diameter, tan in contact with 5% KOH solution, surface with delicately echinulate, length 0.2–0.9 μm, non-starchy, columnar process under scanning electron microscope. Capillitial hyphae up to 1.0–7.0 μm in diameter, thick-walled, brownish yellow, unbranched, wall surface rough, with surface debris. Exoperidium: 747–1198µm thick, The pseudoparenchymatous layer formed of pseudoparenchymatous structured angular cells, 19–41 × 13–29 μm; fibrous layer formed of thick-walled interlacing filament tissue, 3.7–6.6 μm; the mycelium layer formed of thick-walled hyphae, diameter 2.6–5.3 μm.

Additional specimens examined: China, Jilin province: Lushuihe Town, Fusong County, Baishan City, alt. 308 m, 20 August 2021, Lisong Mu, m082013 (HMJAU65728); China, Jilin province: Jilin Agricultural University, Changchun City, alt. 318.1 m, 12 September 2021, X. Wang, WX2191205 (HMJAU65729); China, Jilin province: Jingyuetan National Forest Park, Changchun City, alt. 306 m, 18 September 2021, X. Wang, WX2191808 (HMJAU65730); China, Jilin province: Daweizigou, Hancongling State-Owned Forest Farm, Dunhua City, 27 July 2022, X. Wang, 220727WX160 (HMJAU65731); China, Jilin Province: Lianhua Mountain Primitive Forest Park, Panshi City, alt. 453 m, 8 August 2022, X. Wang, 220808WX193 (HMJAU65732); China, Jilin Province: Luotong Mountain, Liuhe County, Tonghua City, alt.488 m, 11 August 2022, X. Wang, 220811WX211(HMJAU65733); China, Jilin Province: Sanxianjia National Forest Park, Liuhe County, Tonghua City, alt. 666 m, 12 August 2022, Fang Guo, gf22812201 (HMJAU65734), same location, 11 August 2022, X. Wang, 220811WX235 (HMJAU65735); China, Jilin Province: Yuhuangshan, Tonghua City, alt. 521 m, 14 August 2022, X. Wang, 22081410W (HMJAU65736); China, Jilin Province: Qingshan Vanke Resort, Jilin City, alt. 935 m, 15 August 2022, Siying Li, L77 (HMJAU65737); China, Jilin Province: Nanhu Park, Changchun City, alt. 295 m, 17 August 2022, X. Wang, 220817WX253 (HMJAU65738); China, Jilin Province: Changchun Zoo and Botanical Garden, Changchun City, alt. 246 m, 23 August 2022, X. Wang, 2282303W (HMJAU65739).

Habitat: Scattered on the humus layer of *Pinus sylvestris* var. *mongholica* and *Quercus mongolica*.

Distribution: Jilin Province, China.

**Figure 14:**
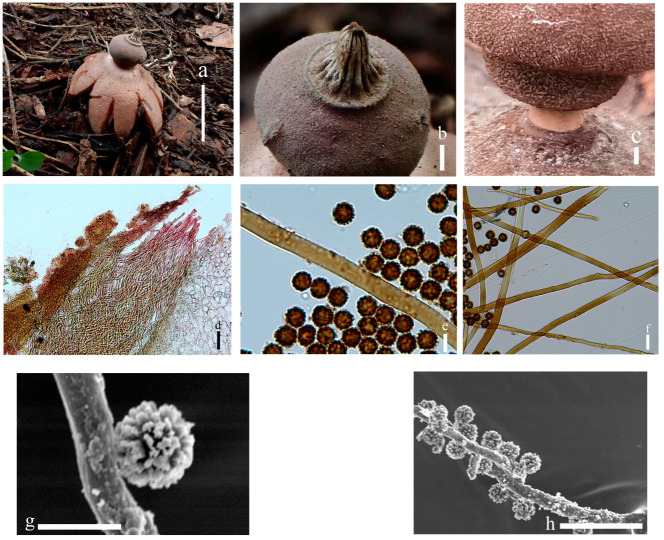
*Geastrum microphole* (HMJAU65718, HMJAU65726), Bars: (**a**) basidiomata = 2 cm; (**b**) peristome = 1 mm; (**c**) stalk = 1 mm; (**d**) exoperidium = 50 μm; (**e**) basidiospores = 5 μm; (**f**) capillitial hyphae = 10 μm; (**g**) = 5 μm; (h) = 10 μm.

**Figure 15:**
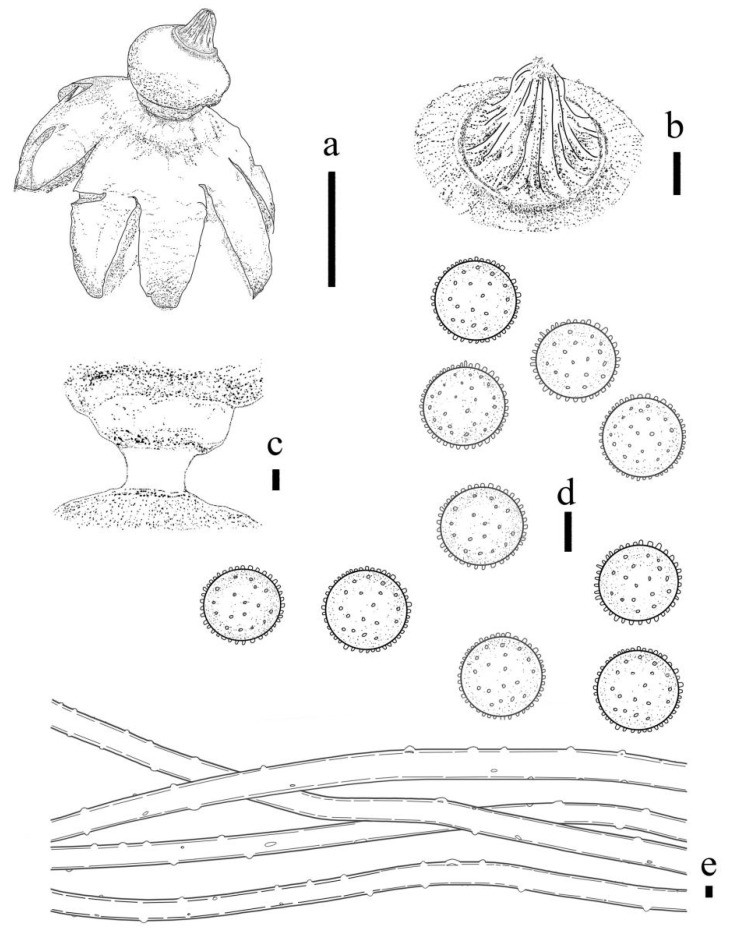
*Geastrum microphole*, Bars: (**a**) basidiomata = 2 cm; (**b**) peristome = 1 mm; (**c**) stalk = 1 mm; (**d**) basidiospores = 2 μm; (**e**) capillitial hyphae = 1 μm.

***Geastrum microphole*** T. Bau & X. Wang, **sp.nov.** (Figure 14 and Figure 15).

MycoBank no: MB846873.

Diagnosis: Differs from *G. berkeleyi* Massee by the peristome, which is sulcate, pseudoparenchymatous layer white, pale brownish to gloom chestnut, a few forming collars at base of stalk [25]. Differs from *G. pseudostriatum* Hollós by the pseudoparenchymatous layer, which is initially greyish pink to pale brownish and later beige-brown to dark brown, peristome sulcate, endoperidium and pseudoparenchymatous layer surface are attached with white frost or white particles [12,13]. Differs from *G. pectinatum* Pers. by the stalk, which has a base with a long stalk, usually over 3.0 mm length, the pseudoparenchymatous layer displays complete retention or partial shedding, with shedding often forming a collar at the base of the stem [26].

Type: China, Jilin Province: Jingyuetan National Forest Park, Changchun City, 43°52′ N, 125°21′ E, alt. 306 m, 18 September 2021, X. Wang, WX2191803 (holotype, HMJAU65720).

Etymology: ‘*microphole*’ refers to its rough endoperidium surface with grey granular protrusions.

Description: Expanded basidiomata 1.2–3.2 cm. Exoperidium: arched, dehiscence often greater than halfway down, at maturity splits into 7–9 lobes, lobes 0.3–1.5 cm wide, extremely narrow at the apex. Pseudoparenchymatous layer: rather thick, rough surface, reddish brown (8D4), purple-tan to tan, contracted along margin of lobes, aseptic collar. Fibrous layer: dull red (8C3), tightly attached to the mycelial layer. Mycelial layer: dark brown (8F8), easily dislodged, encrusted with debris.

Endoperidial body: globular, 0.9–1.5 cm in diameter, projecting apically or extending into a beak, 0.3–0.5 cm length, with an apophysis, stipitate 0.2–0.3 cm more apparent after drying. Endoperidium: dark brown (8F4), grey (8E1) surface rough with grey granular protrusions, for the residual or crystal in the mesoperidum, and greyish villus visible under the dissecting microscope, gleba black. Peristome: broad-conical, sulcus, darker in color than the endoperidium, distinctly protruding peristomal ring.

Basidiospores: spherical, 3.7–5.0 μm in diameter, yellowish brown to dark brown in contact with 5% KOH solution, surface with a delicately warry or short columnar process, length 0.6–0.9 μm, non-starchy, columnar process under scanning electron microscope. Capillitial hyphae up to 1.0–5.0 μm in diameter, thick-walled, brownish-yellow, unbranched, with sparse surface debris. Exoperidium 557.1–660.4µm thick, the pseudoparenchymatous layer formed of the pseudoparenchymatous of a structured angular cell, 5.3–29.2 × 4.1–28.2 μm; fibrous layer formed of thin-walled interlacing filament tissue, 3.1–5.8 μm; the mycelium layer formed of thin-walled hyphae diamater tissue 1.6–5.1 μm.

Additional specimens examined: China, Jilin Province: Baishan Protection Station, Changbaishan District, Baishan City, alt. 811.7 m, 21 August 2021, Fang Guo, g82110 (HMJAU65719); China, Jilin Province: Jingyuetan National Forest Park, Changchun City, alt. 306 m, 26 August 2021, X. Wang, WX2182610 (HMJAU65718), same location, 18 September 2021, X. Wang, WX2191809 (HMJAU65721), 10 October 2021, Lisong Mu, m21101001 (HMJAU65722), 18 August 2022, X. Wang, 22WX2610818 (HMJAU65724); China, Jilin Province: Luotong Mountain, Liuhe County, Tonghua City, alt. 488 m, 11 August 2022, X. Wang, 22WX2130811 (HMJAU65723); China, Jilin Province: Youhao River, Xinli Town, Changchun City, alt. 370 m, 16 September 2022, Fang Guo, gf22916304 (HMJAU65725); China, Jilin Province: Jilin Agricultural University, Changchun City, alt. 318.1 m, 23 September 2022, T. Bau, T092303 (HMJAU65726).

Habitat: Open or semi-exposed to dried humus layer under *Pinus sylvestris* or *Quercus mongolica.*

Distribution: Jilin Province, China.

## 4. Discussion

The phylogenetic placement of the *Geastrum* clades has been discussed by Zamora et al., who found 14 clades within *Geastrum* [11] The new species are distributed in four sections, viz., Sect. *Myceliostroma*, Sect. *Exareolata*, Sect. *Corollina,* and Sect. *Campestria*.

*Geastrum laneum* was clustered with *G. neoamericanum* in our phylogenetic analyses, and can be distinguished through the presence of an encrustation of debris [43]. Morphologically, *G. laneum* resembles *G. mirabile* Mont; they can be distinguished based on differences in the peristome, basidiospore, and whether an encrustation of debris is extant [26]. It differs from *G. laevisporum* J.O. Sousa & Baseia by the mycelial layer, the latter being orange white, densely intermixed with sediments, felted, peeling away in irregular patches with age exposing the fibrous layer, not persistently [44]. It also differs from *G. javanicum* Lév. by the mycelial layer and habitat, the latter encrusted with debris and grown in mixed forest or on sandy soil, as well as a few on stumps; peristome fibrillose [26].

*Geastrum litchi* was clustered with *G. argentinum* in our phylogenetic analyses, and can be distinguished based on whether they have stalks, differences in the peristome, and whether an encrustation of debris is extant [15]. Morphologically, it differs from *G. litchiforme* by the pseudoparenchymatous layer and mycelial layer, the latter distinguished by: frequently forming a collar around the base of the endoperidium, a mycelial layer encrusted with debris, undelimited peristome, an overall dark brown color, being easily separable from the expanding basidiome, and a strong odor of crushed unexpanded basidiomata, similar to that of bok choy [42]. It differs from *G. corollinum* (Batsch) Hollós by the pseudoparenchymatous layer and mycelial layer, the latter orange-brown when fresh and gray-white when dry, encrusted with debris, and the exoperidium, which has strong hygroscopicity [45]. It differs from *G. floriforme* Vittad. by the pseudoparenchymatous layer, the latter not easy to break or fall off, with its nondelimited peristome, and a exoperidium with strong hygroscopicity [46]. 

*Geastrum mongolicum* was clustered with *G.rufescens* and *G. argentinum* in our phylogenetic analyses [13,15]. Morphologically, the three species can be distinguished based on whether they have stalks, on differences in the peristome, and whether an encrustation of debris is extant. *Geastrum mongolicum* is similar in size and morphology to *G. arenarium* Lloyd [12]. All are large species and have stalks, however, the somewhat hygroscopic exoperidial rays, the lack of crystalline matter on the endoperidial surface and smaller spores are distinct in *G.arenarium.*

*Geastrum pseudosaccatum* is a systematic species, which means this species presents no great difference from the control species in macroscopic morphology, but a large branch appeared in the phylogenetic tree. The species was clustered with *G. saccatum* in our phylogenetic analyses [26]. Morphologically, it resembles *G. saccatum*, but the two species can be distinguished based on the degree of fineness of the mycelium layer’s villus. *Geastrum pseudosaccatum* is similar in size and morphology to *G. fimbriatum* Fr. [13]. Both do not have collars, but *G. fimbriatum* mycelial layer is encrusted with debris, undelimited peristome, and it is a widely distributed and well-known species.

*Geastrum melanorhynchum* is similar in size and morphology to *G. morganii* Lloyd, *G. triplex* Jungh. and *G. reticulatumin* Desjardin & Hemmes [42]. All are large species and have unexpanded basidiomatas that are onion-shaped with a distinct papilla. The four species can be readily distinguished by the different texture of the surface of the unexpanded basidiomatas and the structure of the peristome. The *G. morganii* pseudoparenchymatous layer frequently forms a collar, peristome is undelimited, and is irregularly sulcate. *Geastrum reticulatum* has the characteristic reticulated pattern created by lines of raised hyphae. The *G. triplex* with collar and is usually distinctly delimited by a circle of lighter color.

*Geastrum oxysepalum* was clustered with *G. melanorhynchum* in our phylogenetic analyses. Morphologically, the two species can be distinguished based on differences in the peristome and whether felt is present on the mycelial layer surface. *Geastrum oxysepalum* is similar in size and has morphology to *G. velutinum* Morgan and *G. triplex* [45,47]. The mycelium layer is often separated from the fiber layer to form two layers of lobes and peristome is not delimited in *G. velutinum*. *Geastrum triplex* is a widely distributed species with collar and usually distinctly delimited by a circle of lighter color.

*Geastrum microphole* was clustered with *G. pseudostriatum* and *G. berkeleyi* Massee in our phylogenetic analyses. Morphologically, the three species can be distinguished based on differences in the peristome and whether a crystal is present on the endoperidium surface. *Geastrum microphole* is similar to *G. campestre* Morgan and *G. pectinatum* Pers. [12,26]. All are large species and have stalks. The three species can be readily distinguished by the different texture or color of the surface of the pseudoparenchymatous layer, the structure of the peristome and different from the length of the handle. Pseudoparenchymatous layer is found in young pinkish specimens with age brown to grey brown, greyish fibrous layer, grey to grey brown endoperidium, and is distinctly warty in *G. campestre*. The stalk of the G. *pectinatum* is mostly flat and long, 0.3–0.7 cm in diameter.

In this study, through the combination of morphology and molecular data, seven new species of the genus were found in China. It shows that the diversity of forest macrofungi in China is extremely rich (Dai et al. 2021) [48], and it also provides important data, thus supporting the systematic study of the genus in the future. However, there are still many species of *Geastrum* that lack molecular data, which limits the systematic study of this genus [11]. For the time being, the best gene marker for the identification of most *Geastrum* species is ITS, while more terminal nodes in phylogenetic trees need to be investigated by utilizing more gene markers, such as tef1 and RPB1. There are only five tef1 sequences and fifteen RPB1 sequence of *Geastrum* in NCBI (https://www.ncbi.nlm.nih.gov/protein (accessed on 26 November 2022)). It is necessary to obtain more gene fragments to build a more objective phylogenetictree, and therefore, more research needs be carried out in the future.

## Figures and Tables

**Table 1:** The reaction system used in this study.

Designation	Dosage (μL)
DNA template	4.0
Forward primer	1.0
Reverse primer	1.0
2× M5 HiPer plusTaqHiFi PCR mix (with green dye)	12.5
Nuclease-free ddH2O	11.5

**Table 2:** Specimens used in the phylogenetic analysis of *Geastrum* and GenBank accession numbers. The new species are represented in bold.

Species	Geographic	Voucher	GenBank Accessions No.	
	Origin	Number	ITS	nrLSU
*G. albonigrum*	Panama	MA-Fungi 36140-2	KF988349	KF988468
*G. argentinum*	Argentina	MA-Fungi 82605	KF988353	KF988473
*G. argentinum*	Argentina	LPS 48446	KF988352	KF988472
*G.* cf. *arenarium*	Spain	Herb. Zamora 76	KF988338	KF988470
*G.* cf. *arenarium*	Spain	MA-Fungi 68191	KF988350	KF988469
*G. austrominimum*	Australia	MEL 2292062	KP687491	KP687452
*G. austrominimum*	Australia	MEL:2276089	KP687490	KP687451
*G. beijingense*	China	BJTC 248	MZ508872	-
*G. beijingense*	China	BJTC 073	MZ508873	-
*G. benitoi.*	Spain	MA:Fungi 87324	KP687494	KP687455
*G. berkeleyi*	Spain	MA-Fungi 74668	KF988354	KF988474
*G. berkeleyi*	Slovakia	MJ8673/MJ867	KC581985	KC581985
*G. brunneocapillatum*	Brazil	UFRN:Fungos:2286	MH634996	MH635029
*G.* cf. *calceum*	Brazil	UFRN-Fungos 723	KF988340	KF988477
*G. campestre*	USA	MICH 28566	KF988358	KF988480
*G. coronatum*	Hungary	PRM:842868(holo)	KP687495	KP687456
*G. coronatum*	Spain	Zamora 181	KP687496	KP687457
*G. courtecuissei.*	France	LIP:FH 2004090503	MH635003	MH635033
*G. corollinum*	Spain	MA-Fungi 5746	KF988359	KF988481
*G. corollinum*	Sweden	Herb. Sunhede 7744	KF988360	KF988482
*G. dolomiticum*	Hungary: Fejér	FP20150908(holotype)	MT569463	MT569455
*G. dolomiticum*	Hungary: Veszprém	FP20151015	MT569464	MT569456
*G. elegans*	Spain	Herb. Zamora 189	KF988366	KF988488
*G. elegans*	Sweden	UPS F-560810	KF988367	KF988489
*G. fimbriatum*	Spain	Herb. Zamora 234	KF988369	KF988491
*G. fimbriatum*	Sweden	Herb. Sunhede 7739	KF988370	KF988492
*G. flexuosum*	Sweden	UPS F-119844	KF988371	KF988493
*G. floriforme*	Spain	Herb.Zamora 453	KF988373	KF988495
*G. floriforme*	Spain	MA-Fungi 69173	KF988372	KF988494
*G. fornicatum*	Spain	Herb.Zamora 255	KF988374	KF988496
*G. fornicatum*	Spain	MA-Fungi 30749	KF988375	KF988497
*G. fuscogleba*	USA	NY Trappe 9500	KF988377	KF988499
*G. fuscogleba*	USA	NY Trappe 1071	KF988376	KF988498
*G. glaucescens*	Argentina	MA-Fungi 83762	KF988378	KF988500
*G. glaucescens*	Argentina	MA-Fungi 83763	KF988379	KF988501
*G. gorgonicum*	Cape Verde	MA-Fungi 92118	MN754045	MN754083
*G. gorgonicum*	Cape Verde	MA-Fungi 92116	MN754046	MN754084
*G. hansagiense*	Hungary	BP110893	MN582739	MN582739
*G. hansagiense*	Hungary	GBL1	MN582753	-
*G. hariotii*	Agentina	MA-Fungi 83765	KF988381	KF988504
*G. hariotii*	Dominican	MA-Fungi 80070	-	KF988503
*G.* aff. *hariotii*	Brazil	MA-Fungi 78296	KF988382	KF988505
*G. hieronymi*	Argentina	MA-Fungi 83767	KF988344	KF988509
*G. hieronymi*	Agentina	MA-Fungi 83766	KF988384	KF988508
*G. hungaricum*	Czech	Sunhede 5993	KP687500	KP687461
*G. hungaricum*	Spain	Zamora 611	KP687501	KP687462
*G. ishikawae*	Brazil	UFRN Fungos 2785	-	NG.060682
*G. javanicum*	Brazil	UFRN-Fungos 1215	KJ127031	-
*G. kotlabae*	Spain	MA-Fungi 39563	KF988385	KF988510
*G. kotlabae*	Spain	Herb.Zamora 440	KF988386	KF988511
*G. kuharii*	Argentina	MA:Fungi:86913	KP687502	KP687463
*G.* aff. *lageniforme*	Argentina	MA-Fungi 83768	KF988389	KF988516
*G.* aff. *lageniforme*	Niger	COFC Hama 327	KF988390	KF988517
*G.* aff. *lageniforme*	Argentina	MA-Fungi 83770	KF988391	KF988518
*G. lageniforme*	Spain	Herb. Zamora 316	KF988339	KF988514
*G. lageniforme*	Spain	Herb. Zamora 207	KF988388	KF988513
***G. laneum***	**China**	**HMJAU65711**	**OP964640**	**OP964638**
***G. laneum***	**China**	**HMJAU65704 (type)**	**OP964641**	**OP964639**
***G. laneum***	**China**	**HMJAU65705**	**OP964641**	-
***G. litchi***	**China**	**HMJAU65716 (type)**	**OQ360756**	**OP964619**
***G. litchi***	**China**	**HMJAU65717**	-	**OP964620**
*G. marginatum*	Spain	ERRO 2012112609	KP687504	KP687465
*G. marginatum*	Czech	PRM:842884 (holo)	KP687507	KP687468
*G. melanocephalum*	Spain	Herb. Zamora 34	KF988395	KF988522
*G. melanocephalum*	Sweden	Herb. Sunhede 7737	KF988396	KF988523
***G. melanorhynchum***	**China**	**HMJAU65765**	**OP964616**	-
***G. melanorhynchum***	**China**	**HMJAU65764 (type)**	**OP964617**	**OP964614**
***G. melanorhynchum***	**China**	**HMJAU65768**	**OP964618**	**OP964615**
*G. meridionale*	Spain	Herb. Zamora 252 (holo)	KF988412	KF988540
***G. microphole***	**China**	**HMJAU65720 (type)**	**OP964636**	**OP964643**
***G. microphole***	**China**	**HMJAU65721**	**OP964637**	**OP964644**
*G. mirabile*	Japan	TNS:KH-JPN10-714	JN845109	JN845227
*G. minutisporum*	Argentina	CORD15	KM260665	-
*G. minutisporum*	Argentina	CORD14	KM260664	-
*G. minimum*	Sweden	MA-Fungi 86669	KF988405	KF988533
***G. mongolicum***	**China**	**HMJAU65762**	**OP964647**	**OP964645**
***G. mongolicum***	**China**	**HMJAU65763 (type)**	**OP964648**	**OP964646**
*G. morganii*	Canada	Herb. Lebeuf HRL0177 (holo)	KF988406	KF988534
*G. neoamericanum*	Brazil	UFRN:Fungos:2302 (holo)	MH635001	MH635040
*G. neoamericanum*	French	LIP:JLC12030103	MH635014	MH635038
*G. ovalisporum*	Bolivia	MA-Fungi 47184	KF988411	KF988539
*G. ovalisporum*	Argentina	MA:Fungi 86670	-	KP687476
***G. oxysepalum***	**China**	**HMJAU65730**	**OP964629**	-
***G. oxysepalum***	**China**	**HMJAU65735**	**OP964630**	-
***G. oxysepalum***	**China**	**HMJAU65734**	**OP964631**	**OP964621**
***G. oxysepalum***	**China**	**HMJAU65727 (type)**	**OP964632**	**OP964622**
***G. oxysepalum***	**China**	**HMJAU65728**	**OP964633**	**OP964623**
*G. parvistriatum*	Spain	JCZ 272	JN943162	JN939572
*G. parvisporum*	Argentina	BAFC:51926	MG196037	MG196035
*G. parvisporum*	Argentina	MA-Fungi 83793	KF988461	KF988596
*G. pectinatum*	Spain, Lugo	MA:Fungi:28156	KP687516	KP687478
*G. pleosporum*	Cameroon	MA-Fungi 56971	KF988416	KF988544
*G. pouzarii*	Czechoslovakia	MA-Fungi 2944	KF988417	KF988545
*G. pouzarii*	Czechoslovakia	Herb. Sunhede 7494	KF988418	KF988546
*G. pseudostriatum*	Sweden	MJ050919O	KC581990	-
*G. pseudostriatum*	Sweden	MJ8240	KC581991	-
*G. pseudostriatum*	Sweden	MJ7573O1	KC581992	KC581992
*G. pseudostriatum*	Sweden	BP 22110	NR132884	
*G. pseudolimbatum*	Spain	Herb. Zamora 231	KF988419	KF988547
*G. pseudolimbatum*	Sweden	UPS F-560804	KF988420	KF988548
***G. pseudosaccatum***	**China**	**HMJAU65778**	**OP964624**	-
***G. pseudosaccatum***	**China**	**HMJAU65781**	**OP964625**	**OP964635**
***G. pseudosaccatum***	**China**	**HMJAU65772**	**OP964626**	-
***G. pseudosaccatum***	**China**	**HMJAU65774**	**OP964627**	-
***G. pseudosaccatum***	**China**	**HMJAU65769 (type)**	**OP964628**	**OP964634**
*G. quadrifidum*	Spain	Zamora 300	KP687524	KP687486
*G. quadrifidum*	Sweden	MA86671	KF988422	KF988550
*G. rubropusillum*	Brazil	UFRN:Fungos:2308	MH634994	MH635027
*G. rubellum*	France	LIP:CL/MART 8067B	MH635009	-
*G. rubellum*	Brazil	UFRN:Fungos:2844	MH634999	MH635031
*G. rufescens*	Spain	Herb. Zamora 253	KF988424	KF988552
*G. rufescens*	Spain	Herb. Zamora 274	KF988425	KF988553
*G. rusticum*	Brazil	UFRN Fungos 1217	-	NG060634
*G. saccatum*	Japan	UPS F-530056	KF988428	KF988558
*G. schweinitzii*	Argentina	MA-Fungi 83779	KF988437	KF988567
*G. schmidelii*	Sweden	UPSF-560805	KF988435	KF988565
*G. schmidelii*	China	HMAS 84118	MZ50883	MZ509381
*G. setiferum*	Baseia	MA-Fungi 83781	-	KF988571
*G. setiferum*	Argentina	MA-Fungi 83782	-	KF988572
*G. smardae*	Spain	Herb. Zamora 527	KF988441	KF988574
*G. smardae*	Canada	Herb. Lebeuf HRL 0160	KF988440	KF988573
*G. striatum*	Sweden	MA-Fungi 86672	KF988443	KF988577
*G.* spc070607	China	HMJAU65787	-	OP967186
*G.* spc70808	China	HMJAU65788	-	OP967185
*G.* spc70906	China	HMJAU65790	OP972576	-
*G.* sp22DQ21	China	HMJAU65789	OP972575	OP967188
*G.* spQWQ8767	China	HMJAU65786	-	OP967183
*G.* spQWQ155634	China	HMJAU65785	-	OP967187
*G. tenuipes*	Australia	CANB:775658	KP687527	KP687489
*G. tenuipes*	Australia	CANB:738350	KP687526	KP687488
*G. thanatophilum*	USA	MICH 72012	KF988364	KF988486
*G. triplex*	Madagascar	UPS F-014630-213863	KF988444	KF988578
*G. triplex*	Argentina	MA-Fungi 83784	KF988445	KF988579
*G. velutinum*	China	BJTC 598	MZ508877	-
*G. violaceum*	Agentina	MA-Fungi 82487	KF988451	KF988586
*G. violaceum*	Agentina	BAFC 51671	KF988450	KF988585
*G. yanshanense*	China	BJTC 381	MZ508878	MZ509383
*G. yanshanense*	China	BJTC 057	MZ508879	MZ509384
*Sphaerobolus iowensis*	USA	SS11	AY487958	AY439014
*S. iowensis*	Indiana	SS1	AY487950	-
*Schenella pityophila*	Spain	Herb. Zamora 530	KF988346	KF988464
*S. pityophila*	Spain	Herb. Zamora 531	KF988347	KF988465

Note: “-” means no relevant genetic information, and the new species are represented in bold.

## Data Availability

All the sequences have been deposited in GenBank (https://www.ncbi.nlm.nih.gov (accessed on 16 March 2022)) and Mycobank (https://www.mycobank.org (accessed on 16 March 2022)); The matrices of the trees have been uploaded to TreeBASE (http://www.treebase.org (accessed on 27 December 2022); accession number S29978).
